# The function of p53 and its role in Alzheimer’s and Parkinson’s disease compared to age-related macular degeneration

**DOI:** 10.3389/fnins.2022.1029473

**Published:** 2022-12-21

**Authors:** Peter Wolfrum, Agnes Fietz, Sven Schnichels, José Hurst

**Affiliations:** Centre for Ophthalmology, University Eye Hospital Tübingen, Tübingen, Germany

**Keywords:** p53, neurodegeneration, Alzheimer’s disease, Parkinson’s disease, retina, age-related macular degeneration

## Abstract

The protein p53 is the main human tumor suppressor. Since its discovery, extensive research has been conducted, which led to the general assumption that the purview of p53 is also essential for additional functions, apart from the prevention of carcinogenesis. In response to cellular stress and DNA damages, p53 constitutes the key point for the induction of various regulatory processes, determining whether the cell induces cell cycle arrest and DNA repair mechanisms or otherwise cell death. As an implication, aberrations from its normal functioning can lead to pathogeneses. To this day, neurodegenerative diseases are considered difficult to treat, which arises from the fact that in general the underlying pathological mechanisms are not well understood. Current research on brain and retina-related neurodegenerative disorders suggests that p53 plays an essential role in the progression of these conditions as well. In this review, we therefore compare the role and similarities of the tumor suppressor protein p53 in the pathogenesis of Alzheimer’s (AD) and Parkinson’s disease (PD), two of the most prevalent neurological diseases, to the age-related macular degeneration (AMD) which is among the most common forms of retinal degeneration.

## Introduction

Neurodegeneration describes pathological processes leading to the malfunctioning and destruction of nerve cells. With life expectancies steadily increasing, neurodegenerative diseases are a growing concern for public health (Deuschl et al., 2020). While some of the most frequent diseases concerning the brain are Alzheimer’s (AD) and Parkinson disease (PD), also retinal-related degenerative diseases such as the age-related macular degeneration (AMD) are increasingly prevalent (Checkoway et al., 2011; Li et al., 2020). The retina is an anatomical extension of the brain where certain parallels can be drawn since it is as well derived from the neuroectoderm and part of the central nervous system (CNS). Therefore, neurodegenerative diseases of the brain and the retina share characteristic damages to nerve tissue, which causes a significant loss in the self-reliance and quality of life for patients. As the diseases progress, patients can lose important functions of the nervous system or in the case of the retina, their eyesight. Although the causes of neurodegenerative diseases in general are multifactorial, some diseases are known to have a strong hereditary component (Armstrong, 2020). For instance, the early onset familial Alzheimer’s disease (EOFAD) represents a subgroup of AD, whereby mutations in certain loci promote pathogenetic pathways leading to an early onset, additional neurological symptoms as well as a more severe course of disease (Wu et al., 2012). To date, the therapeutic possibilities in neurodegenerative diseases are insufficient. While PD is often progressive during treatment, the symptoms of diseases like AD can only be slightly alleviated by therapy and some forms of AMD even offer no medical therapy option at all. The therapeutic challenge results from different circumstances like pharmacokinetic problems related to the blood brain- and blood retinal-barrier, as well as diverse disease trigger factors and the poorly understood complexity of the pathogeneses (Modi et al., 2010; Duraes et al., 2018). The goal for all neurodegenerative diseases is to find novel therapeutical approaches that can prevent or decelerate the progression of these conditions. Understanding the underlying molecular and pathological mechanisms is essential for the development of such treatments. The tumor suppressor protein p53 has been shown to constitute a key point in the emergence of carcinosis (Lane, 1994). Lately, p53 has also been linked to the pathogenesis of non-cancerous diseases, which is why this review focuses on the influence of p53 in the pathogenesis of neurodegenerative diseases (Szybinska and Lesniak, 2017). Thus, in the following, the function of p53 in AD, PD and AMD is outlined and a comprehensive overview of the current state of research is offered.

## The tumor suppressor protein p53

p53 was first discovered in 1979 and was named after its molecular mass of 53 kilodaltons (kDa) (Levine and Oren, 2009; Soussi, 2010). p53 acts as a transcriptional factor and is composed of the N-terminal Domain (NTD), the folded DNA-binding Domain (DBD) and tetramerization Domain (TD), as well as the regulatory Domain (RD), all together contributing to its functioning (Ho et al., 2006). The NTD thereby interacts with and activates enzymes of the transcription machinery. The DBD facilitates the process of DNA binding, together with the TD which is responsible for the tetrameric quaternary structure formation of p53 (Chene, 2001). The RD further stabilizes the tetrameric formation and is involved in the regulation of other proteins, whereby not all functions are completely resolved yet (Ho et al., 2006; Romer et al., 2006; Retzlaff et al., 2013). The correct conformation of p53 is crucial. Therefore, changes in the confirmation can lead to structural aberrations and possibly p53-unfolding accompanied by alterations of its functioning. Besides different conformational states, 12 different p53 protein-isoforms exist, which originate from alternative processes of splicing, initiation of translation or promoter activation (Khoury and Bourdon, 2011).

**FIGURE 1:**
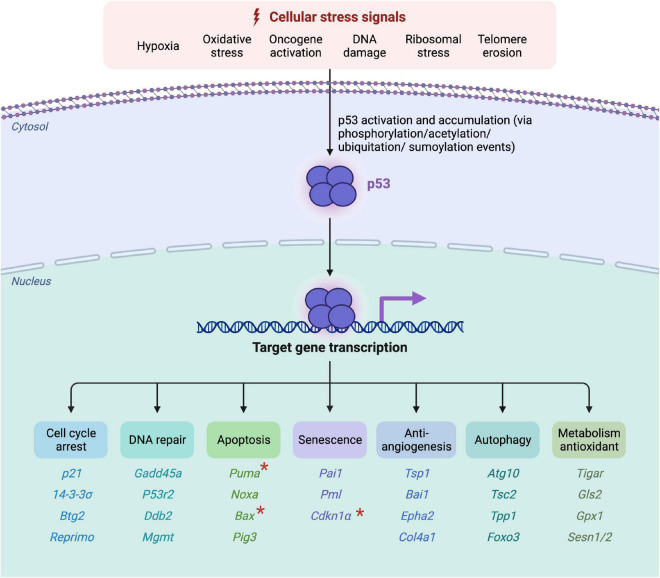
Overview of p53-activation, target gene induction and its corresponding functioning. Active oncogenes or the accumulation of cellular damages and stressors lead to the phosphorylation, acetylation, ubiquitation or sumoylation of p53 *via* the activation of kinases and transcriptional factors. Following its activation, p53 binds to its corresponding DNA-promoter-regions, through which cell cycle arrest, DNA repair mechanisms, apoptosis, senescence, anti-angiogenic, autophagic or antioxidant metabolism mechanisms are induced. The corresponding target genes are listed below the according function. The “*” marked genes represent neurodegenerative p53-influenced downstream targets, discussed in this review (Created with BioRender.com).

In more than 50% of all human cancer diseases, p53 is found to be mutated, which points out its importance in the prevention of tumor genesis (Joerger and Fersht, 2008). Besides that, ongoing research has discovered its relevance for the regulation of numerous processes in the cell. In response to cellular damages, post-translational-modifications (PTM) of p53 take place, which lead to a decreased binding affinity to the inhibitory MDM2 protein as well as an increased target gene expression. In the case of mild stress, p53 activates pathways resulting in cell cycle arrest and DNA-repair mechanisms, whereas following irreparable damages, apoptosis or senescence is initiated (Figure 1; Kastenhuber and Lowe, 2017). Senescence has been linked to both physiological and pathological conditions, the latter including ageing, cancer, and other age-related disorders (Munoz-Espin and Serrano, 2014). Furthermore, it has also been shown that p53 and the levels of its downstream targets are tissue specific (Burns et al., 2001; Tanikawa et al., 2017). Through different alterations such as mutations or following excessive amounts of oxidative stress, p53 mediated responses can also lead to the emergence of diseases.

## p53 activation and target gene induction

To prevent possible toxic consequences under normal conditions, the basal concentration of p53 is low and regulated by the negative feedback loop of the ubiquitin ligase MDM2, which is the cellular antagonist of p53 and contributes to the critical step in mediating p53 degradation by nuclear and cytoplasmic proteasomes (Moll and Petrenko, 2003). The activation of oncogenes like the Ras oncogene as well as the accumulation of cell and DNA damages, which further lead to the induction of different kinases and transcriptional factors like *P300/CB* or *PCAF*, initiate modifications of p53 *via* phosphorylation and acetylation (Steegenga et al., 1996; Prives and Manley, 2001; Reed and Quelle, 2014). These changes lead to an increase in the DNA-binding capacity, through its DBD, as well as a reduced binding of MDM2 (Shieh et al., 1997; Kastenhuber and Lowe, 2017). Furthermore, the half-life value as well as its transcriptional activity can also be influenced by other chemical reactions like ubiquitation or sumoylation, which thereby lead to modifications of certain lysine residues in the TD and RD (Romer et al., 2006). The two reactions are again in response to cellular stress and are facilitated by the ubiquitination-proteasome system as well as proteins of the small ubiquitin-like modifier family (SUMO) (Seeler and Dejean, 2003; Li et al., 2022). Besides the four named PTMs, numerous other protein modifications exist which again lead to changes of p53-functioning (Clark et al., 2022).

Once p53 is activated, it binds *via* its DBD to the consecutive DNA-Promotor sequences of its target genes. Through the binding of one DBD dimer, consisting of two DBD monomers, the tetrameric p53-DNA-complex is formed (Romer et al., 2006). Depending on which promoter sequence p53 has bound to, different downstream genes are expressed. Thereby again, depending on various factors, the p53 mediated signal-transduction can either lead to the survival of the cell *via* cell cycle arrest and consecutive DNA repair mechanisms, the permanent cell cycle arrest *via* senescence or the lethal cell death *via* apoptosis (Mijit et al., 2020). Besides these functions, p53 can also induce anti-angiogenic, autophagic or antioxidant metabolism processes (Figure 1; Shu et al., 2007; Riley et al., 2008). Additional functions of p53 are also investigated, such as an influence in cell fate determination, as well as an impact in the regulation of the necrotic cell death (Crighton et al., 2006; Rozan and El-Deiry, 2007; Ying and Padanilam, 2016). Due to the large size of the protein, it is likely that even more unknown functions are also regulated by p53.

## p53-mediated apoptosis regulation

The most common p53-mediated apoptosis mechanisms are the intrinsic and the extrinsic apoptosis pathway which are mainly responsible for cell death in mammalian cells (Wang et al., 2015; Aubrey et al., 2018). In the intrinsic pathway, increased levels of p53 lead to the upregulation of the pro-apoptotic BH3-only proteins and the inhibition of anti-apoptotic proteins of the BCL-2-family. This causes increased BAX and BAK levels and further initiates the mitochondrial membrane permeabilization, Apoptosome formation and Caspase activation as part of the mitochondrial apoptotic pathway (Aubrey et al., 2018; Xu et al., 2019).

**FIGURE 2:**
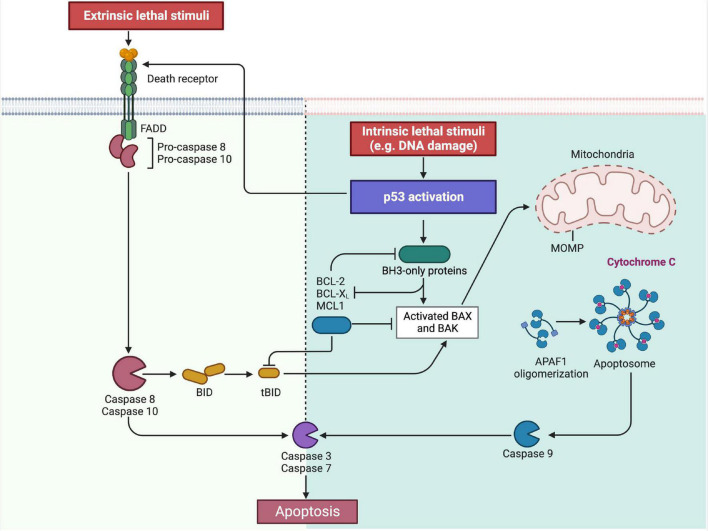
Overview on the intrinsic and extrinsic apoptosis pathway. As part of the intrinsic pathway, the activation of p53 leads to the increased expression of the BH3-only proteins, which is followed by BAX and BAK. Simultaneously the BH3-only proteins also inhibit the anti-apoptotic proteins BCL-2, BCL-X, and MCL1. This then leads to the permeabilization of the outer mitochondrial membrane (MOMP) of the mitochondria with the consequence of cytochrome c release, apoptosome formation and caspase 9-activation. Contrary, the same anti-apoptotic proteins BCL-2, BCL-X and MCL1 inhibit the BH3-only proteins under physiological conditions in order to prevent apoptosis induction. In the extrinsic pathway, p53 can upregulate so called “death receptors” in the cell membrane. Following the binding of the respective ligand, the death signal is triggered and transmitted into the cell. This then leads to the activation of caspase 8 and 10. Both pathways finally lead to the induction of caspases 3 and 7, through which the apoptosis is irreversible induced. In addition, the extrinsic and intrinsic apoptosis pathways are also connected by the BH3 interacting domain death agonist (BID), allowing a secondary activation of the intrinsic pathway (Created with BioRender.com).

In regards to the extrinsic pathway, the activation of p53 can induce an upregulation of the death receptor density in the cell membrane (Carneiro and El-Deiry, 2020). The binding of certain ligands on these receptors, like the Fas-ligand, marks the induction of the extrinsic apoptosis pathway. Both pathways eventually lead to the activation of Caspases 3 and 7, initiating irreversible cell death. Moreover, the BH3 interacting domain death agonist (BID) is a protein that connects the extrinsic to the intrinsic apoptosis pathway which demonstrates that interferences between the two pathways can also occur (Aubrey et al., 2018; Figure 2).

## p53 and neurodegeneration

In general, symptoms of brain-related neurodegenerative disease, like AD or PD, emerge through the decline of neuronal cells. Elevated levels of p53, cohering with neurodegeneration, have previously been observed, implying a key role of p53 in the pathogeneses of brain-related neurodegeneration (Chang et al., 2012). As a response to specific events such as DNA damage, metabolic compromises or increased oxidative stress, p53 can trigger apoptosis in neuronal cells. Thereby, depending on its stimulus, p53 induces the cell death either through the induction of the classical extrinsic and intrinsic apoptosis pathway, or through a synapses-specific cell death mechanism, whereby the apoptosis is directly triggered at the mitochondrial level, without an upstream transcriptional activity (Culmsee and Mattson, 2005).

## Influence of p53 on Alzheimer’s disease

**FIGURE 3:**
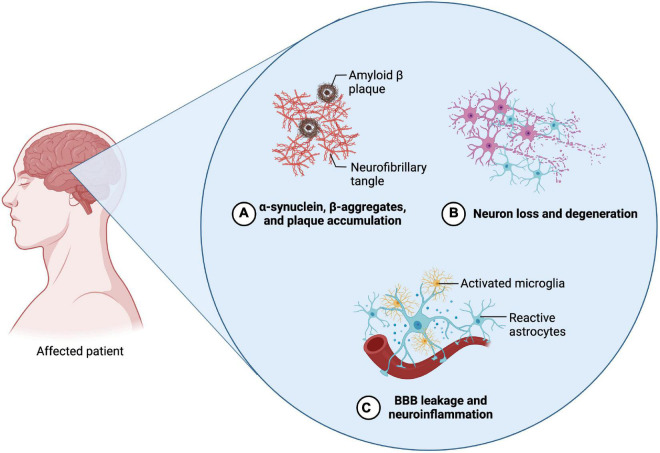
Neuropathological features of Parkinson’s disease (PD) and Alzheimer’s disease (AD). The two diseases exhibit similar neuropathological features. **(A)** The emergence of abnormal protein depositions can thereby be observed during the initial phases of both diseases. In regard to AD, β-amyloid plaques and Tau protein accumulations can be found, whereas in PD α-synuclein aggregates. **(B)** The abnormal accumulation then leads to the induction of apoptosis and the decline of the according neuronal cells. Regarding AD, primarily neurons in the area of the cortex and the hippocampus are affected. In PD, mainly dopaminergic neurons in the substantia nigra are affected **(C)** Alongside the progressive neuronal cell decline, neuroinflammation and the disruption of the blood-brain barrier can be observed, which is accompanied by their pathognomonic symptoms (Created with BioRender.com).

Alzheimer’s disease is a progressive disease affecting the cortex cerebri and hippocampus and is leading to neuronal cell loss and cerebral atrophy. As the disease progresses, cognitive impairments such as dementia, behavioral changes and disorientation symptoms occur. The pathogenesis of the accompanying neurodegeneration has been extensively studied and is associated with several events leading to β-amyloid (Aβ) plaque formation and deposition, as well as the accumulation of hyperphosphorylated Tau (p-tau) and further neurofibrillary tangle formation (Figure 3; Chang et al., 2012; Dos Santos Picanco et al., 2018).

As investigated in multiple studies, patients affected by AD exhibit elevated levels of p53 in several sections of their brain (de la Monte et al., 1997; Kitamura et al., 1997; Szybinska and Lesniak, 2017). One of the assumed factors leading to a p53-dependent apoptosis induction in neuronal cells is the accumulation of Aβ (Troy et al., 2000). In presence of Aβ, increased levels of the pro-apoptotic BAX, as well as decreased levels of anti-apoptotic BCL-2 proteins have been observed *in vitro*, as well as in post mortem brain analysis of AD affected patients (Paradis et al., 1996; Su et al., 1997; Chang et al., 2012). Moreover, a link between Aβ and an increase in the p53 upregulated modulator apoptosis protein (PUMA), which is another BH3-only protein that inhibits anti-apoptotic BCL-2 proteins, has been observed (Feng et al., 2015). Fogarty et al. detected that the treatment of cortical neurons with Aβ leads to the phosphorylation of p53 at serine 15, which is a well-known PTM of p53, leading to its activation and an increased transcriptional activity (Fogarty et al., 2010; Loughery et al., 2014). In the same study, an alternative cell death mechanism, operated by the process of lysosomal membrane permeabilization was observed. Thereby, the association of phosphorylated p53 with the lysosomal membrane leads to changes in its integrity and further the leakage of hydrolases into the cytosol. Subsequently, cell death is initiated, either through direct cell death, *via* the digestion of vital proteins, or indirectly *via* a caspase cascade (Boya and Kroemer, 2008; Fogarty et al., 2010).

Another nouveau theory about the role of p53 in AD pathogenesis is about the unfolded p53 modification. Uberti et al. (2002, 2008) discovered conformational changes of p53 in fibroblasts of AD affected patients (Abate et al., 2020). The thereby observed and later named “unfolded p53” protein comprises structural aberrations which can be induced through various influences. In the review of Clark et al., different factors, like reactive oxygen species (ROS), reactive nitrogen species (RNS), Aβ-amyloid or metallothionein are listed which lead to the unfolded p53 modification in context of AD pathogenesis (Clark et al., 2022). Since under its normal composition, p53 controls many processes in the cell in order to avoid cellular damages, the emergence of unfolded p53 subsequently initiates abnormal regulations like the inhibition of the SOD and GAP-43 pathway or the promotion of the CD44 and mTOR pathway, overall leading to neuronal dysfunction and the promotion of neurodegeneration (Abate et al., 2020).

One more recent finding in AD pathogenesis is the formation of p53 oligomers and fibrils followed by its colocalization with the Tau protein, which was shown in AD affected brains (Farmer et al., 2020). It is assumed that these aggregative changes of p53 lead to an endogenous p53 seeding in neurons. Furthermore, a subsequently altered p53 function, referable to the mislocalization of phosphorylated p53 outside of the nucleus is thereby also hypothesized, to be responsible for the progressive neurodegeneration in AD pathogenesis (Farmer et al., 2020).

## Influence of p53 on Parkinson’s disease

The loss of dopamine secreting neurons, as well as the accumulation of Lewy bodies, which constitute of misfolded α-synuclein, are the main events characterizing the pathogenesis of PD (Figure 3; Balestrino and Schapira, 2020). Patients affected by the disease experience hyperkinesia, which is accompanied with akinesis, rigor, tremor, as well as postural instability. In the further course of the disease, it can also lead to cognitive impairments and dementia (Beitz, 2014).

Different cellular stress factors such as mitochondrial dysfunction, oxidative stress or an abnormal protein aggregation have been associated with the pathogenesis of PD (Naoi and Maruyama, 1999; Luo et al., 2022). Since p53-induced cell death occurs also in response to cellular stress and p53 is able to induce cell death in dopaminergic neurons, it thus seem likely that it is involved in the pathogenesis of PD (Shu et al., 2007; Lu et al., 2017). Mogi et al. observed elevated levels of p53 as well as an increase of apoptosis-related proteins like BAX in examined parkinsonian brains (Mogi et al., 2007; Liu et al., 2019). Furthermore, phosphorylated p53 modifications have also been detected in tissue of the substantia nigra in PD affected patients (Martire et al., 2015). Research on proteins of the BCL-2 family suggest a central role in the embryological development of dopaminergic neurons as well as its loss in disease like PD. Thereby, as part of the embryological related cell death, apoptotic processes such as the caspase 3 induced DNA fragmentation, were observed in mice-models (Jackson-Lewis et al., 2000). Additionally, the overexpression of anti-apoptotic BCL-2 proteins preserves dopaminergic neurons from toxin-induced apoptosis and leads to an increased number of dopaminergic cells postnatally (Jackson-Lewis et al., 2000; Liu et al., 2019).

Unfolded p53 protein has not been observed in PD affected patients (Abate et al., 2020). Nevertheless, a different modification of p53 has been detected in the pathogenesis of PD: 1-methyl-4-phenyl-1,2,3,6-tetrahydropyridine (MPTP) induces the cell death of dopaminergic neurons in the substantia nigra of mice which has ever since been used as model for the investigation of PD pathogenesis (Nicotra and Parvez, 2002). Mandir et al. (2002) observed that following the MPTP treatment, p53 was poly(ADP-ribosy)lated by the Poly(ADP-ribose)-Polymerase 1 (PARP). These changes thereby lead to the stabilization of p53 as well as an increase in its levels (Martire et al., 2015). During the initial phase of extensive p53-ribosylation, it was further observed that the DNA binding of p53 was inhibited. Therefore, only after the following deribosylation of p53, the binding of the according DNA target gene regions is enabled again, which finally leads to the apoptosis-induction of dopaminergic neurons (Mandir et al., 2002).

## Influence of p53 in the age-related macular degeneration

**FIGURE 4:**
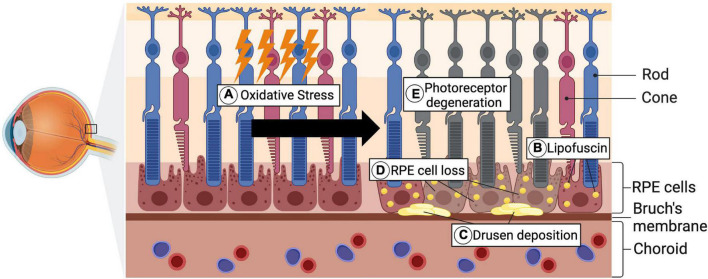
Pathological hallmarks of AMD pathogenesis. **(A)** As a consequence of genetic predispositions, environmental factors, as well as aging, oxidative stress accumulates. **(B,C)** Lipofuscin, which is composed of lipid- and protein residues, forms under increased oxidative stress levels and further accumulates in RPE cells. Alongside the lipofuscin, Drusen depositions, consisting of extracellular material, form in the area of Bruch’s membrane. **(D)** Through the progredient accumulation of these depositions, the RPE cell death is subsequently triggered. **(E)** Secondary this then leads to photoreceptor degeneration and occurrence of visual impairments (Created with BioRender.com).

AMD is a degenerative, progressive disease leading to photoreceptor degeneration in the area of the *macula lutea* and constitutes the leading cause for vision loss in elderly people from industrialized countries (Loss et al., 2018). In the development of AMD, there is a causal relationship between oxidative stress and the pathogenesis of the disease (Yildirim et al., 2011; Schnichels et al., 2021). In early disease stages, lipid deposits also called lipofuscin and drusen accumulate in the area of Bruch’s membrane and RPE cells (Figure 4; Bowes Rickman et al., 2013). Subsequently, the accumulation leads to the functional impairment of RPE cells and thus their cell death (Mitchell et al., 2018). One of the main functions of RPE cells is the support of photoreceptor cells, hence its loss secondarily leads to photoreceptor degeneration, which is accompanied by vision impairment (Strauss, 2005; Mitchell et al., 2018). While patients affected by early forms of AMD, are mostly asymptomatic or experience slight visual function impairments reading in the dark, advanced disease stages can lead to severe vision loss (McLeod et al., 2009; Bowes Rickman et al., 2013). The later stages of AMD can further be sub-classified into the “*dry*” and the “*wet*” form. Whereas dry AMD is also referred to as atrophic AMD and accounts for 90% of all cases of AMD, the wet form is associated with neovascularization processes and coheres with rapid vision loss (Bowes Rickman et al., 2013; Akyol and Lotery, 2020). Since the RPE cell loss and consequent photoreceptor degeneration are the crucial hallmarks of AMD pathogenesis, the cell death of both cell types has been extensively investigated.

The expression of p53 in various ocular tissues, including multiple retinal layers in transgenic mice and rats, have previously been observed suggesting an overall function of p53 in the eye (Shin et al., 1999; Vuong et al., 2012). p53 is associated with retinal responses to radiation and oxidative stress. Miller et al. investigated photoreceptor cell death mechanisms by treating cells of the 661W-photoreceptor cell line with the oxidative stressor CI-1010. Elevated levels of the p53-induced caspases 3 and 8 were thereby detected without an increase of cytochrome c, suggesting a p53 dependent, non-mitochondrial cell death mechanism in photoreceptors (Miller et al., 2006). Contrary, *in vivo* studies from Lansel et al. and Marti et al. documented increased apoptosis induction in mice from extensive light exposure, independent of a p53 prevalence. In both studies, p53-wild-type mice and p53 null-mice were treated with bright light exposure treatments, following electroretinogram (ERG) and morphological analyses. Subsequently after the exposure, both mice types showed similar ERG-, as well as histochemical- and biochemical- results, suggesting that the light induced apoptosis in photoreceptors is not p53-dependent (Lansel et al., 1998; Marti et al., 1998; Vuong et al., 2012). However, increased p53-dependent apoptosis rates were observed in bright light exposed human ARPE-19 cells. The apoptosis rate was further only increased under the premise that components of the RPE lipofuscin pigment were present, which again is a concomitant factor in AMD pathogeneses (Westlund et al., 2009; Vuong et al., 2012). Furthermore, Bhattacharya et al. observed an increased basal rate of the p53-dependent apoptosis in aged human RPE cells, which could be contiguous with the progressive cell death in AMD pathogenesis. The increased p53 activity was attributed to the acetylation and phosphorylation of p53, which again leads to the loss of the negative feedback loop function of MDM2 (Bhattacharya et al., 2012). As mentioned earlier, these two modifications of p53 constitute two of the most common PTMs increasing the p53 activity levels (Aubrey et al., 2018). In conjunction with the increased p53 activity, a reduction of the anti-apoptotic BCL-2 proteins was thereby measured as well, supporting the thesis of a p53 dependent cell death (Bhattacharya et al., 2012).

## Discussion

Alzheimer’s disease is the most common form of dementia and has many parallels with the pathogenesis of AMD. Thereby, both diseases primarily have an onset in elderly people and exhibit similar pathogenetic risk factors, such as smoking tobacco, hypertension or hypercholesterolemia (Armstrong, 2019; Heesterbeek et al., 2020). The cardinal features of AD include the extracellular accumulation of Aβ as well as the intracellular deposition of p-tau. Neuroinflammation and brain iron dyshomeostasis accompany Aβ and p-tau depositions and together lead to progressive neuronal cell death and dementia. In AMD patients, inflammatory processes as well as the accumulation of Aβ in drusen has also been observed, suggesting an overlapping pathology (Anderson et al., 2004). On the other hand, the pathogenesis of PD also shares several similarities with AMD. For both diseases, an increased incidence in elderly people, inflammatory processes and oxidative stress seem to play a major role (Chen et al., 2021). In a study that was conducted by Choi et al., the association of AMD with AD and PD was investigated. They show that patients affected by AMD exhibit an increased risk for also developing AD or PD, providing evidence for a linkage between retinal- and brain-related neurodegenerative diseases (Choi et al., 2020).

**FIGURE 5:**
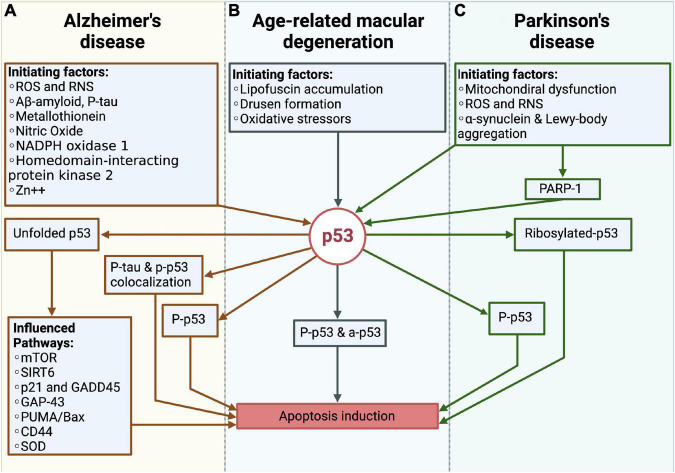
Overview over the role of p53 in Alzheimer’s disease (AD), Parkinson’s disease (PD) and the age-related macular degeneration (AMD). **(A)** In AD, different pathogenic factors like e.g., reactive oxygen species (ROS) and reactive nitrogen species (RNS), Aβ or p-tau, promotes the formation of unfolded p53, phosphorlyated-p53 (p-p53) as well as the colocalization of the tau protein and p-p53. Through these changes, the apoptosis of neurons is induced. **(B)** In AMD, the lipofuscin accumulation and drusen formation as well as oxidative stressors like e.g., the blue light induced ROS formation, initiate phosphorylated and acetylated p53, which further leads to the cell death of RPE cells. **(C)** In PD, mitochondrial dysfunction, ROS production and the accumulation of pathogenic proteins like α-synuclein and Lewy-bodies influence p53. Together with PARP-1 these factors promote the ribosylation and phosphorylation of p53, which is responsible for the cell death of dopaminergic neurons.

The before mentioned findings on p53 in AD, PD and AMD strongly suggest that the protein is critical for the pathogeneses of the three diseases (Figure 5). Whereas to date, the exact role of p53 in AMD pathogenesis is still unclear, much more is known about its pathogenesis in AD and PD.

In AD and PD, the direct observation of elevated p53 levels, together with the increase in pro-apoptotic BCL-2 proteins are coherent with the occurring cell death. Vice versa, increased protein levels of anti-apoptotic BCL-2 proteins seem to promote survival of the cells, which again supports the thesis of a p53-dependency. In summary, it seems likely that in AD and PD the mitochondria-dependent apoptosis is one of the assumed forms of cell death, leading to neurodegeneration. Yet, it is to expect that cell death is also initiated through further p53-dependent and independent mechanisms, like the previously described p53 dependent lysosomal membrane permeabilization (Fogarty et al., 2010). Furthermore, different PTMs have been observed for both diseases (Figure 5). The observation of unfolded p53, which is initiated by a variety of factors was only observed in AD patients, suggesting that the according structural changes are AD-specific (Abate et al., 2020). Otherwise, PARP-1 appears to be one of the main factors in PD, being responsible for p53-ribosylation and altering its functioning in the context of PD. One similar observation was the detection of phosphorylated p53, which was observed in both pathogeneses.

In the pathogenesis of AMD, the previously described *in vivo* and *in vitro* studies by Lansel et al. (1998); Marti et al. (1998); Miller et al. (2006) analyzing the associated photoreceptor degeneration, show contradictory results concerning the role of a p53-related photoreceptor cell death. One reason for the opposing results could be related to the fact that in the compared studies different cellular stressors were used, with the possibility of not every stressor leading to a p53-dependent apoptosis. Another cause could be the 661W cone photoreceptor cell line, which was used by Miller et al. and does not represent the actual *in vivo* situation (Miller et al., 2006). In terms of the RPE cell death findings, a p53-dependent cell death seems more likely. The described study by Bhattacharya et al., evidencing an increase in the p53 dependent apoptosis in human RPE cells, outlines a first indication of a p53-dependent cell death (Bhattacharya et al., 2012). Therefore, it is possible that in the AMD pathogenesis only the RPE cell death is p53 dependent. As with AD and PD, phosphorylated-p53 levels were found, whereas besides acetylated-p53, which constitutes a similar p53 modification, other PTMs have not yet been identified in AMD pathogenesis (Figure 5).

Nevertheless, the overall current state of research about the p53 dependent neurodegeneration of the retina is insufficient and further research needs to be done. For instance, the less investigated Müller cells, which have been shown to be essential for the survival of photoreceptors as well, potentially also undergo a p53-dependent apoptosis and could strengthen the link of a p53-dependent AMD pathogenesis (Dubois-Dauphin et al., 2000). If this link is further validated, new therapeutic treatment approaches could benefit from such findings. For example, a novel therapy approach could be the modulation of the p53-related pro- and anti-apoptotic BCL-2 family proteins, which is already investigated in the treatment of cancerous diseases (Thomas et al., 2013; Perini et al., 2018; Wei et al., 2020). A similar projection for the future treatment of neurodegenerative diseases has previously been described by Shacka and Roth (2005).

## Conclusion

Even though p53 was discovered over 40 years ago, research about its functioning is conducted to this day. With the ongoing exploration and the growing number of p53-disease-associations outside of cancerous affections, the protein p53 is more important than ever. Altogether, the current literature strongly suggests connections between brain and retinal related neurodegenerative diseases. Besides the occurrence of the phosphorylated p53 modification, which has been detected in all three diseases, further specific modifications in AD and PD have been observed. Especially the unfolded p53 was thereby specific to AD and could potentially function as a biomarker in the future. In contrast, less is known about the role of p53 in the pathogenesis of AMD, with the current findings suggesting only a p53 dependent cell death in RPE cells and not in photoreceptor cells. More research is needed to further proof a function of p53 in the pathogenesis of AMD.

## Author contributions

PW, JH, and SS devised the project. PW conceptualized and wrote the manuscript with input from JH, SS, and AF. JH, SS and AF revised the manuscript. All authors provided critical feedback and contributed to the final manuscript.
